# A comprehensive multi-directional exploration of phytochemicals and bioactivities of flower extracts from *Delonix regia* (Bojer ex Hook.) Raf., *Cassia fistula* L. and *Lagerstroemia speciosa* L.

**DOI:** 10.1016/j.bbrep.2020.100805

**Published:** 2020-09-11

**Authors:** Faisal Bin Rahman, Sium Ahmed, Priya Noor, Mir Md. Mahbubur Rahman, S.M. Azimul Huq, Md. Taharat Elahi Akib, Abdullah Mohammad Shohael

**Affiliations:** Cell Genetics and Plant Biotechnology Laboratory, Department of Biotechnology and Genetic Engineering, Jahangirnagar University, Savar, Dhaka, 1342, Bangladesh

**Keywords:** Bioefficacy, Flowers, Folk medicine, Mice model, Ornamental plants, Phytoconstituents, aPTT, Activated partial thromboplastin time, CE, Catechin equivalent, CfFME, *Cassia fistula* flower methanolic extract, DPPH, 2,2-diphenyl-1-picrylhydrazyl, DrFME, *Delonix regia* flower methanolic extract, GAE, Gallic acid equivalent, IC50, Half-maximal inhibitory concentration, LsFME, *Lagerstroemia speciosa* flower methanolic extract, PT, Prothrombin time, SEM, Standard error of the mean, TAE, Tannic acid equivalent, TFC, Total flavonoid content, TPC, Total phenolic content, TTC, Total tannin content, UV, Ultra-violet

## Abstract

*Delonix regia* (Bojer ex Hook.) Raf., *Cassia fistula* L. and *Lagerstroemia speciosa* L. are three ornamental plants that produce colorful flowers. The present study aimed to evaluate the phytochemicals and bioactivities of methanolic extracts of flowers from *Delonix regia* (DrFME), *Cassia fistula* (CfFME), and *Lagerstroemia speciosa* (LsFME). The presence of ten different chemical classes in varying degrees was confirmed while qualitatively screened. During quantitative determination, LsFME possesses the highest amount of total phenolic (418.0 mg/g), flavonoid (50.8 mg/g), and tannin (256.3 mg/g) contents. The extracts showed excellent antioxidant capacity in a concentration-dependent manner with the lowest IC50 value (41.51 μg/mL) displayed by LsFME. LsFME paralyzed the experimental worms at 2.95 min and killed at 3.96 min. DrFME was found to be more effective in thrombolytic (35.5% clot lysis) and anticoagulant activities. Negligible hemolytic activity (IC50 > 200 μg/mL) found for all extracts which suggest their less potential toxicity. The *in vivo* experiments revealed that the CfFME has the highest analgesic (64.34% pain inhibition) activity while LsFME has the highest antidiarrheal (70.27% inhibition) and antihyperglycemic (46.94% inhibition) activities at 400 mg/kg of body weight doses. This study has shown the presence of phytochemicals and potential bioactivities which indicates the possibility of these flowers to be used as a source of phytochemicals as well as safe and effective natural medicine.

## Highlights

• Krisnachura (*Delonix regia*), Sonalu (*Cassia fistula*), and Jarul (*Lagerstroemia speciosa*) are colorful flower producing ornamental plants grown in Bangladesh.
• The flowers of these plants are rich in phytochemicals and exhibit antioxidant, anthelmintic, thrombolytic, anticoagulant, analgesic, antidiarrheal and antihyperglycemic activities as determined by *in vitro* and *in vivo* methods.
• The flowers can be used as a source of safe and effective natural medicine.

## 1 Introduction

Plants are the natural reservoir of various medicinal agents and thus, they have been used to cure different diseases and disorders for thousands of years [1]. Medicinal plants are extensively studied as a source of safe and effective medicine because they possess bioactive compounds (secondary metabolites) which are mainly responsible for different healing effects. Various bioactive components are present in the medicinal plants, which are the key source of new pharmaceuticals and health care products [2]. These compounds are preferable as they are natural in origin and have fewer side effects than synthetic compounds. Moreover, many synthetic compounds are produced through the influence of natural compounds [3]. As a result, a good number of modern drugs have been isolated or originated from the bioactive components of natural sources and mainly influenced by their implication as traditional medicine [4]. Due to the side effects of synthetic drugs, demands of natural remedies are increasing worldwide which has motivated researchers to search for different plant parts with their potential activities.

*Delonix regia* (Bojer ex Hook.) Raf., known as Krisnachura in Bangladesh is a species of flowering plants, belonging to the family of Fabaceae. It is also known as flame tree as it has beautiful red flowers. The tree is a native to Madagascar and widely planted as ornamental trees in many countries of the tropical region [5]. The flowers traditionally been used to treat constipation, inflammation, rheumatoid arthritis, diabetes, pneumonia, and malaria [6]. The leaf extracts of this plant have been reported to be used as antimicrobial, antioxidant, antihyperglycemic, anti-inflammatory, cytotoxic and hepatoprotective agent [5,[7], [8], [9]]. The stem bark extracts were found to have antioxidant and antimicrobial properties [10,11].

*Cassia fistula* L. is a member of Fabaceae family and known as Sonalu in Bangladesh. It is native to India and also grown in Bangladesh, Mauritius, South Africa, Mexico, Brazil, China, Nepal, West Indies, and East Africa. It has beautiful yellow flowers that's why it is grown as an ornamental plant [12]. The plant extracts have been used in the treatment of skin diseases, inflammatory diseases, rheumatism, anorexia, and jaundice. Bark extracts from this plant have anti-inflammatory and antioxidant activities [13]. Leaf extracts of these plants have been implicated as antibacterial, antifungal, hepatoprotective, wound healer, and hypoglycemic [[14], [15], [16], [17]]. The pod extracts have been reported as an antioxidant [18]. It's flower extracts have also been investigated as an anti-aging, antibacterial, and antifungal agents [19,20].

*Lagerstroemia speciosa* L., belonging to the family Lythraceae which is commonly known as Jarul in Bangladesh. It is a medicinal and ornamental tree native to China and cultivated in tropical and subtropical regions including Bangladesh, India, Malaysia, Thailand, Philippines, Indonesia, and Japan for its beautiful purple flowers. The leaves are traditionally being used as folk medicine for anti-fibrotic, antioxidant, anti-inflammatory, anti-diabetic, and anti-obesity [[21], [22], [23], [24]]. The bark extracts have also been reported as antinociceptive [25]. The root extracts have been reported to show analgesic and antidiarrheal activities [26]. The flower extract have been investigated for antioxidant, antimicrobial, and anti-aging potentials [27,28].

Those flowering plants are not only ornamental but also have significant medicinal values in terms of a versatile range of efficacies. Most of the previous research were aimed to explore the biological activities in leaves and other plant parts. The utilization of flowers as a source of phytochemicals and bioactivities was not well reported. That's why the present study aimed to investigate the phytochemicals and bioactivities of the flower extracts from three plants using *in vitro* and *in vivo* methods.

## 2 Materials and methods

### 2.1 Study location and plant materials

The experiments were conducted at the Cell Genetics and Plant Biotechnology Laboratory (CGPBL), Department of Biotechnology and Genetic Engineering, Jahangirnagar University, Dhaka-1342, Bangladesh (23°53′14″ N 90°15′56″ E). Flowers of Krisnachura (*Delonix regia)* Sonalu *(Cassia fistula),* and Jarul (*Lagerstroemia speciosa)* were used as the experimental materials in this study. About 1.0 kg of each flower was collected from the Jahangirnagar University campus during May 2019.

### 2.2 Experimental animals and human blood samples

Four to five weeks aged male Swiss albino mice having bodyweight of 20–30 g (Maintained by the Department of Pharmacy, Jahangirnagar University, Savar, Dhaka, Bangladesh) were used for the analgesic, antidiarrheal, and antihyperglycemic experiments. Before initiating the experiments, the animals were kept under standard environmental conditions, maintained at 55%–65% relative humidity and exposure to alternative 12:12 h light and dark cycle at an ambient temperature of 26 ± 2 °C. Proper supply of foods and water ad libitum was ensured. This study was approved by the Biosafety, Biosecurity, and Ethical Clearance Committee, Jahangirnagar University (Memo no.: BBEC, JU/M 2020 (3)4).

During the collection of human blood samples, 5.0 mL of whole blood (vein) was drawn from 10 young (21–26 years old) and healthy human (male) volunteers without a history of oral contraceptive or anticoagulant therapy using a protocol approved by the Biosafety, Biosecurity, and Ethical Clearance Committee of Jahangirnagar University [Memo no.: BBEC, JU/M 2020 (3)2]. An earlier consent form was signed by the volunteers for the collection of blood samples from them.

### 2.3 Preparation of extracts

The collected flowers were washed with distilled water, sun-dried for seven days, and then dried in a hot air oven (JSR, Korea) at 50 °C for 72 h. The dried flowers were powdered in a mechanical grinder. The powdered materials were taken in conical flasks with 70% methanol as the solvent and kept them in an orbital shaker for three days at room temperature. The extracts were filtered through Whatman no. 1 filter paper and the filtrates were concentrated using an evaporator at 45 °C, and finally, stock solutions of the extracts (100 mg/mL) were prepared using 0.1 N NaCl.

### 2.4 Qualitative screening of phytochemicals

The freshly prepared crude extracts were qualitatively tested for the presence of secondary metabolites such as alkaloids, carbohydrates, coumarins, glycosides, flavonoids, phenols, resins, saponins, tannins and terpenoids by following the methods described in previous literatures [29,30].

### 2.5 Quantitative determination of total phenolic, flavonoid, and tannin content

The total phenolic content of the extracts was estimated according to the method described previously [31]. Briefly, 0.1 mL aliquots of extracts and standards were mixed with 2.5 mL deionized water followed by the addition of 0.1 mL (2 N) Folin–Ciocalteu reagent. They were mixed well and allowed to stand for 6 min before 0.5 mL of 20% sodium carbonate (Na_2_CO_3_) solution was added. The color was developed after 30 min of incubation at room temperature in the dark. The absorbance was measured at 760 nm in a UV–visible spectrophotometer (T60 UV–Visible Spectrophotometer, PG Instruments Ltd., United Kingdom). Total phenolic content was expressed as milligrams of Gallic Acid (C_7_H_6_O_5_) Equivalent (mg GAE)/g of extract.

Total flavonoid content was determined following the method described previously [31]. Briefly, 0.25 mL of extracts or (+)-catechin standard solution was mixed with 1.25 mL of distilled water, followed by the addition of 0.75 mL of 5% sodium nitrite solution. After 6 min, 0.15 mL of 10% aluminum chloride (AlCl_3_) solution was added, and the mixture was allowed to stand for a further 5 min and then 0.5 mL of 1 M sodium hydroxide (NaOH) was added. The mixture was brought to 2.5 mL with distilled water and mixed well. The absorbance was measured immediately at 510 nm in a T60 UV–visible spectrophotometer. The concentration of flavonoids was expressed as milligrams of Catechin (C_15_H_14_O_6_) Equivalent (mg CE)/g of extract.

The total tannin content was determined using the Folin-Ciocalteu reagent as described previously [29]. Briefly, 0.1 mL of the samples and the standard was added with 7.5 mL of distilled water. Then 0.5 mL of Folin-Ciocalteu reagent and 1.0 mL of 35% sodium carbonate (Na_2_CO_3_) solution were added. The total volume was adjusted to 10.0 mL by adding distilled water. The mixture was incubated at room temperature for 30 min and the absorbance was measured at 725 nm in a T60 UV–visible spectrophotometer. The concentration of tannin was expressed as milligrams of Tannic Acid (C_76_H_52_O_46_) Equivalent (mg TAE)/g of extract.

### 2.6 Determination of antioxidant capacity

Antioxidant capacity was evaluated by the DPPH (2,2-diphenyl-1-picrylhydrazyl) free radical scavenging activity according to the method described previously [32]. Different concentrations (800, 400, 200, 100, 50, 25, 12.5, 6.25 μg/mL) of extracts were dissolved in methanol. 3.0 mL of 0.004% methanol solution of DPPH was added to each test tube. The mixture was incubated for 30 min in dark condition. The absorbance was measured at 517 nm in a UV–visible spectrophotometer. The percent inhibition activity was calculated from [(A_C_-A_S_)/A_C_] × 100, where A_C_ is the absorbance of the control, and A_S_ is the absorbance of the sample. The inhibition curves were prepared and the half-maximal inhibitory concentration (IC50) values were calculated by using non-linear regression analysis in Graphpad Prism 6.0.

### 2.7 In vitro anthelmintic assay

The anthelmintic activity was performed according to the method described previously [33]. The experiments were carried out using aquarium worm (*Tubifex tubifex*) due to its anatomical resemblance with the human intestinal roundworm parasites. They were collected from a local aquarium shop located at Kataban, Dhaka, Bangladesh. Five different concentrations of extract (50, 25, 12.5, 6.25, 3.125 mg/mL) were used for the experiment. Levamisole (Etrax®, ACI Limited, Bangladesh) at a concentration of 1.0 mg/mL was used as a positive control and distilled water was used as a negative control. The experiment was carried out in small beakers (50 mL). Five mL of extracts were taken in each beaker. For every concentration of each extract, three beakers were taken as biological replicates. In each beaker, ten worms were placed. The time was noted using a stopwatch. Two different times such as time for paralysis and time for death was noted. The mean time for paralysis (min) was noted when the movement was stopped except when the worms were shaken vigorously; the time of death (min) was recorded after the confirmation of no sign of movement when shaken vigorously or placed in warm water.

### 2.8 In vitro thrombolytic activity

*In vitro* thrombolytic activity was performed according to the method described previously [34]. In the previously weighed Eppendorf tubes, 500 μL of human blood was transferred. The tubes were incubated at 37 °C for 45 min to form the clot. After clot formation, serum was completely removed without disturbing the clot, and each tube having a clot was again weighed to determine the clot weight. A volume of 100 μL of extracts of different concentrations (5 and 10 mg/mL) were added to each Eppendorf tube containing pre-weighed clot. As a positive control, 100 μL of streptokinase and as a negative control, 100 μL of distilled water were separately added to the tubes. All the tubes were then incubated at 37 °C for 90 min and observed for clot lysis. After incubation, fluid released was removed and tubes were again weighed to observe the difference in weight after clot disruption. The difference obtained in weight taken before and after clot lysis was expressed as the percentage of clot lysis.

Percent clot lysis (%)= (Weight of the released clot/ Clot weight) × 100

### 2.9 In vitro anticoagulant activity

The anticoagulant activity was determined through prothrombin time (PT) test and activated partial thromboplastin time (aPTT) test using methods described previously with slight modification [35].

In this study, prothrombin time (PT) was measured according to Thromboplastin LI kit by Agappe diagnostics Switzerland GmbH and activated partial thromboplastin time (aPTT) was measured according to aPTT kit by Analyticon Biotechnologies AG, Germany. Nine parts of blood and one part of 3.2% trisodium citrate (Na_3_C_6_H_5_O_7_) solution (0.109 M) were mixed gently in sterile 15 mL falcon tubes. The tubes were centrifuged immediately for 15 min at 3000 rpm to obtain platelet-poor plasma. 100 μL of plasma was placed into the test Eppendorf tubes at 37 °C and incubated for 3 min. Then 100 μL extracts of different concentrations were added to the tubes. 200 μL pre-warmed (37 °C) PT reagent was added into each of the test Eppendorf tubes. A timer was started simultaneously and the clotting time in seconds was recorded. For aPTT, 50 μL plasma was taken into each of the test Eppendorf tubes. 50 μL extracts of different concentration was added to the test Eppendorf tubes. The test Eppendorf tubes were subjected to incubation at 37 °C for 2 min. Then pre-warmed (37 °C) 50 μL Calcium Chloride (CaCl_2_) (0.025 M) was added to each of the test Eppendorf tubes. A timer was started simultaneously and the clotting time in seconds was recorded.

### 2.10 In vitro hemolytic assay

The hemolytic assay was conducted according to the method described previously with slight modification [36]. Five mL of blood was centrifuged immediately after collection. The plasma was discarded and the cells were washed three times with phosphate-buffered saline (PBS-pH 7.4) by centrifugation at 1500 rpm for 5 min. The cell suspension was prepared by finally diluting the pellet to 0.5% in PBS. A volume of 2.0 mL of the cell suspension was mixed with 2.0 mL of extracts of various concentrations. The mixtures were incubated for 30 min at 37 °C and centrifuged at 1500 rpm for 7 min. The free hemoglobin in the supernatants was measured spectrophotometrically at 412 nm. PBS and distilled water were used as minimal and maximal hemolytic controls. The level of percent hemolysis by the extracts was calculated according to the following formula:

Hemolysis (%) = (A_t_-A_n_ / A_c_-A_n_) × 100

Where A_t_ is the absorbance of the test sample; A_n_ is the absorbance of the minimal hemolytic control (PBS) and A_c_ is the absorbance of the maximal hemolytic control (distilled water).

### 2.11 In vivo analgesic study (acetic acid-induced writhing test)

Analgesic activity was evaluated by the test of abdominal writhing induced by acetic acid in mice as described previously [37]. A total of fourty Swiss albino mice was taken in each test and fasted for 18 h with free access to water. Each test was performed thrice. The mice were divided into eight groups, containing five mice in each group. Group 1 and 2 received two different doses of DrFME (200 mg/kg and 400 mg/kg body weight) respectively. Group 3 and 4 received two doses of CfFME (200 mg/kg and 400 mg/kg body weight) respectively. Whereas Group 5 and 6 received two doses of LsFME (200 mg/kg and 400 mg/kg body weight) respectively. Group 7 received standard drug (Aspirin at 200 mg/kg body weight). Group 8 was treated with distilled water (10 mL/kg body weight), which served as negative control. After 45 min of respective treatment, each mouse was injected intraperitoneally with 0.7% (v/v) acetic acid at a dose of 10 mL/kg body weight. 15 min later of the injection, the number of writhing responses of each mouse was counted for a 5 min period. To calculate the percentage of inhibition of writhing, the following formula was used:

Inhibition (%) = [1− {No. of writhing (positive control or extracts) / No. of writhing (negative control)}] × 100.

### 2.12 In vivo antidiarrheal study (castor oil-induced diarrheal test)

The antidiarrheal activity was tested according to the method described previously [38]. Forty Swiss albino mice were taken in each test and fasted for 18 h with free access to water. Each test was performed thrice. The mice were divided into eight groups, containing five mice in each group. Group 1 and 2 received two different doses of DrFME (200 mg/kg and 400 mg/kg body weight) respectively. Group 3 and 4 received two doses of CfFME (200 mg/kg and 400 mg/kg body weight) respectively. Whereas Group 5 and 6 received two doses of LsFME (200 mg/kg and 400 mg/kg body weight) respectively. Group 7 received standard drug (Loperamide hydrochloride at 3.0 mg/kg body weight). Group 8 was treated with distilled water (10 mL/kg body weight), which served as negative control; After 30 min of the administration, all mice received 0.5 mL of castor oil orally to initiate diarrhea and were individually placed in cages on blotting paper. At every hour, the paper was changed. During an observation period of 5 h, the number of diarrheal feces was recorded and the percentage of inhibition of defecation was calculated for every group of animals by the following formula:

Inhibition (%) = [1− {No. of diarrheal feces (positive control or extracts) / No. of diarrheal feces (negative control)}] × 100.

### 2.13 In vivo antihyperglycemic study (oral glucose tolerance test)

The antihyperglycemic property was determined as per the procedure previously described [39]. Briefly, fourty Swiss albino mice were taken in each test and fasted for 18 h with free access to water. Each test was performed thrice. The mice were divided into eight groups, containing five mice in each group. Group 1 and 2 received two different doses of DrFME (200 mg/kg and 400 mg/kg body weight) respectively. Group 3 and 4 received two doses of CfFME (200 mg/kg and 400 mg/kg body weight) respectively. Whereas Group 5 and 6 received two doses of LsFME (200 mg/kg and 400 mg/kg body weight) respectively. Group 7 received standard drug (Glibenclamide at 10 mg/kg body weight). Group 8 was treated with distilled water (10 mL/kg body weight), which served as a negative control. All substances were orally administered. Following a period of 1 h, all mice were orally administered 2.0 g glucose/kg of body weight. Blood samples were collected 120 min after the glucose administration by puncturing the heart. Blood glucose levels were measured by using a glucometer (GlucoLeader™ Enhance, HMD BioMedical Inc., Taiwan).

### 2.14 Statistical analysis

All data were displayed as the mean ± standard error of the mean (SEM) for at least three independent biological replications. The statistical analyses were performed using GraphPad Prism 6.0 for Windows (GraphPad Software, USA).

## 3 Results

### 3.1 Qualitative screening of phytochemicals

Qualitative screening generally gives an idea about what type of bioactive compounds are present or absent in the tested extracts. The results obtained from the qualitative screening are given in Table 1. From the present study, it was evident that all three flower extracts were found rich in diverse phytochemicals. The extracts were shown to be positive for all chemical types tested where the degree of presence varied. In DrFME, carbohydrates and coumarins were detected in high quantities. In CfFME, carbohydrates and resins were detected in high quantities. And in LsFME, flavonoids, phenols, and tannins were detected in high quantities.

**Table 1:** Qualitative phytochemical screening of methanolic extracts of flowers from *Delonix regia* (DrFME), *Cassia fistula* (CfFME), and *Lagerstroemia speciosa* (LsFME).

Phytochemicals	DrFME	CfFME	LsFME
Alkaloids	++	++	++
Carbohydrates	+++	+++	++
Coumarins	+++	+	+
Flavonoids	++	++	+++
Glycosides	++	++	++
Phenols	++	++	+++
Resins	+	+++	++
Saponins	+	+	+
Tannins	++	++	+++
Terpenoids	+	++	++

**Indication:** +++ = High; ++ = Moderate; + = Low; - = Not detected.

### 3.2 Quantitative determination of total phenolic, flavonoid, and tannin content

**Fig. 1:**
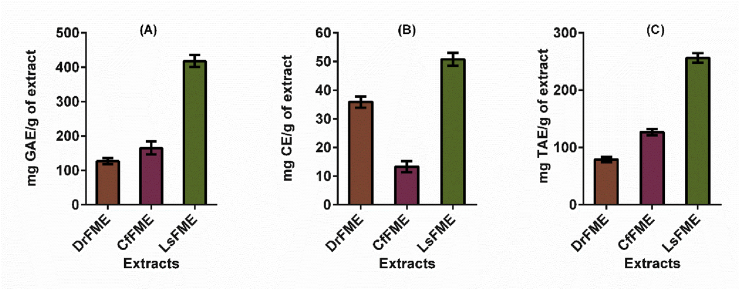
Quantitative determination of (A) total phenolic content; (B) total flavonoid content and (C) total tannin content in methanolic extracts of flowers from *Delonix regia* (DrFME), *Cassia fistula* (CfFME), and *Lagerstroemia speciosa* (LsFME).

Quantitative determination was done for total phenolic, flavonoid, and tannin content and the results are displayed in Fig. 1. In these investigations, LsFME was found to be rich in phenolic (418.0 mg/g), flavonoid (50.8 mg/g) and tannin (256.3 mg/g) content. For total phenol content (Fig. 1A), the order of the extracts in terms of higher to lower quantity was LsFME > CfFME > DrFME. For total flavonoid content (Fig. 1B), the order of the extracts in terms of higher to lower quantity was LsFME > DrFME > CfFME. And for total tannin content (Fig. 1C), the order of the extracts in terms of higher to lower quantity was LsFME > CfFME > DrFME.

### 3.3 Determination of antioxidant capacity

**Fig. 2:**
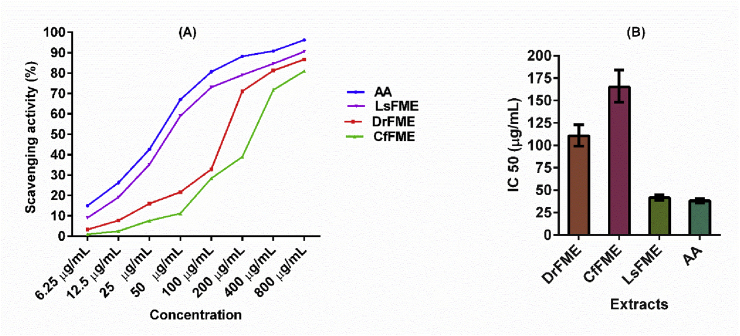
Antioxidant activity of methanolic extracts of flowers from *Delonix regia* (DrFME), *Cassia fistula* (CfFME), and *Lagerstroemia speciosa* (LsFME) in terms of (A) DPPH free radical scavenging assay and (B) half-maximal inhibitory concentration (IC50) of DrFME, CfFME, LsFME and Ascorbic Acid (AA) obtained by non-linear regression analysis at 95% confidence interval.

The antioxidant capacity was determined in terms of DPPH free radical scavenging potentials and shown in Fig. 2. The extracts showed excellent antioxidant capacity where the highest antioxidant capacity was found for LsFME. The antioxidant capacity can be interpreted as LsFME > DrFME > CfFME (Fig. 2A). The half-maximal inhibitory concentration (IC50) values were obtained where it was found that the LsFME (41.51 μg/mL) has the lowest IC50 value, followed by DrFME (110.5 μg/mL) and CfFME (165.0 μg/mL) (Fig. 2B). In contrast, Ascorbic acid was used as a standard which showed an IC50 value of 38.09 μg/mL (Fig. 2B).

### 3.4 In vitro anthelmintic activity

*In vitro* anthelmintic activity was measured by the ability of extracts to paralyze and kill the experimental worms. The results of anthelmintic activity are given in Table 2. The effects were implicated for five different extract concentrations and it is clear from the result that all the extracts showed anthelmintic activity in a concentration-dependent manner. The lowest time for paralysis (2.95 ± 0.13 min) and time of death (3.96 ± 0.05 min) were recorded for LsFME at 50 mg/mL concentration. At 50 mg/mL extract concentration, the CfFME required 3.27 ± 0.13 min to paralyze the worms and 4.46 ± 0.05 min to kill the worms. Besides, the DrFME showed its paralysis effect at 3.55 ± 0.15 min and killing effect at 4.78 ± 0.30 min of extract administration. In contrast, the positive control (Levamisole) paralyzed the worms at 4.26 ± 0.13 min and killed them at 7.15 ± 0.18 min at its 1 mg/mL concentration while there was no sign of paralysis and death in the negative control (distilled water).

**Table 2:** *In vitro* anthelmintic activity of methanolic extracts of flowers from *Delonix regia* (DrFME), *Cassia fistula* (CfFME), and *Lagerstroemia speciosa* (LsFME).

Extracts	Concentration (mg/mL)	Time taken for paralysis (min)	Time taken for death (min)
**DrFME**	50.0	3.55 ± 0.15	4.78 ± 0.30
	25.0	9.02 ± 0.43	11.80 ± 0.83
	12.5	33.06 ± 2.10	52.64 ± 2.06
	6.25	62.28 ± 2.71	90.16 ± 2.38
	3.125	95.39 ± 1.13	130.77 ± 2.86
**CfFME**	50.0	3.27 ± 0.13	4.46 ± 0.05
	25.0	6.68 ± 0.39	10.15 ± 0.38
	12.5	13.48 ± 1.38	20.50 ± 1.99
	6.25	26.95 ± 0.85	36.88 ± 1.10
	3.125	46.00 ± 1.15	53.70 ± 0.90
**LsFME**	50.0	2.95 ± 0.13	3.96 ± 0.05
	25.0	5.70 ± 0.72	9.44 ± 0.26
	12.5	12.24 ± 0.67	18.95 ± 1.07
	6.25	24.63 ± 0.58	33.92 ± 0.76
	3.125	34.97 ± 1.10	48.32 ± 1.17
**Positive control (Levamisole)**	1.0	4.26 ± 0.13	7.15 ± 0.18
**Negative control (Distilled water)**	0	0	0

### 3.5 In vitro thrombolytic activity

**Fig. 3:**
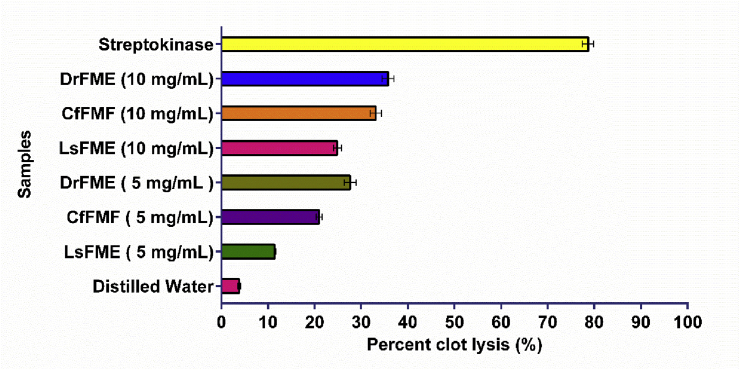
The thrombolytic activity of methanolic extracts of flowers from *Delonix regia* (DrFME), *Cassia fistula* (CfFME), and *Lagerstroemia speciosa* (LsFME).

The thrombolytic activity was evaluated by measuring the ability of the extracts to lyse blood clots and represented by percent clot lysis (%). The results of the thrombolytic activity are depicted in Fig. 3. The extracts were utilized at two different concentrations. Among the extracts, the highest thrombolytic activity (35.5%) was found for DrFME at its 10 mg/mL concentration which was followed by 32.2% thrombolytic activity of CfFME at its 10 mg/mL concentration. The thrombolytic activity of DrFME at its 5 mg/mL concentration (27.8%) was higher than the thrombolytic activity shown by the CsFME at its 10 mg/mL concentration (25.2%). In contrast, the positive control streptokinase showed 78.67% clot lysis activity while the negative control distilled water achieved clot lysis to a negligible extent (3.83%).

### 3.6 In vitro anticoagulant activity

To explore the action in the plasma coagulation cascade, citrated human plasma clotting time was analyzed. The results of anticoagulant activity are displayed in Table 3. The results were shown in terms of prothrombin time (PT) test and activated partial thromboplastin time (aPTT) test. All three extracts were utilized in three different concentrations and found to be effective as anticoagulants as they extended the time of coagulation at different concentrations. However, comparatively, DrFME was found to be more effective because of providing greater anticoagulant activity. In the case of PT, the standard reference time of clotting is 12-15 s. After adding the extracts the time extends to 41 s for DrFME, followed by 35 s for LsFME and 32 s for CfFME at their 1.0 mg/mL extract concentration. In the case of aPTT, the standard reference time of clotting is 25-39 s. After adding the extracts the time extends to 50 s for DrFME, followed by 48 s for LsFME and 42 s for CfFME at their 1.0 mg/mL extract concentration.

**Table 3:** *In vitro* anticoagulant activity of methanolic extracts of flowers from *Delonix regia* (DrFME), *Cassia fistula* (CfFME), and *Lagerstroemia speciosa* (LsFME) through prothrombin time (PT) activated partial thromboplastin time (aPTT) tests.

Extracts/Samples	Concentrations	Prothrombin time (s)	Activated partial thromboplastin time (s)
Reference time for normal blood		10–15	25–39
Sample without extracts		13 ± 0.70	26 ± 0.70
DrFME	1.0 mg/mL	41 ± 0.70	50 ± 1.41
	0.5 mg/mL	36 ± 1.41	41 ± 0.70
	0.25 mg/mL	31 ± 2.12	37 ± 1.41
CfFME	1.0 mg/mL	32 ± 1.41	42 ± 0.70
	0.5 mg/mL	28 ± 0.00	36 ± 2.12
	0.25 mg/mL	24 ± 0.70	31 ± 0.70
LsFME	1.0 mg/mL	35 ± 1.41	48 ± 1.41
	0.5 mg/mL	29 ± 0.70	41 ± 0.70
	0.25 mg/mL	24 ± 2.12	36 ± 2.12

### 3.7 In vitro hemolytic assay

**Fig. 4:**
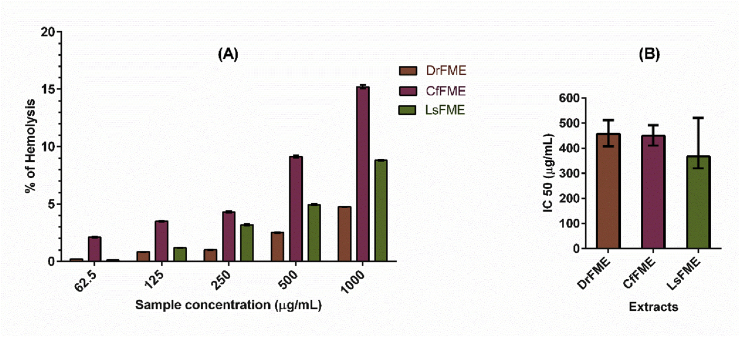
Hemolytic activity of methanolic extracts of flowers from *Delonix regia* (DrFME), *Cassia fistula* (CfFME), and *Lagerstroemia speciosa* (LsFME). (A) The percent hemolysis showed by the extracts at different concentrations. (B) Half-maximal inhibitory concentration (IC50) of DrFME, CfFME and LsFME obtained by non-linear regression analysis at a 95% confidence interval.

Hemolytic activity of different extracts is expressed in percent hemolysis (Fig. 4). The extracts were utilized in five different concentrations and the effects were achieved in a concentration dependent manner. Highest hemolytic activities were found for CfFME at its 1.0 mg/mL (15.21%) and 0.5 mg/mL (9.15%) concentrations followed by LsFME at its 1.0 mg/mL (8.80%) concentration. The IC50 values were obtained as LsFME (366.6 μg/mL) < CfFME (449.1 μg/mL) < DrFME (457.1 μg/mL).

### 3.8 In vivo analgesic study (acetic acid-induced writhing test)

In the acetic acid-induced writhing test, all the experimental mice displayed writhing after the administration of acetic acid. The results are represented in Table 4. After administration of the extracts the reduction of writhing served as a basis where the best activity was shown by the CfFME at the 400 mg/kg body weight dose where the number of writhing was 14.4 ± 2.6 and the inhibition was 64.34%. This was very close to the inhibition (74.72%) observed after the treatment with aspirin which showed the lowest number of writhing compared to any other treatment groups with extracts. Among the other treatments with DrFME (400 mg/kg body weight) and LsFME (400 mg/kg body weight) showed inhibition (%) which are (51.20%) and (32.63%) respectively.

**Table 4:** *In vivo* analgesic activity of methanolic extracts of flowers from *Delonix regia* (DrFME), *Cassia fistula* (CfFME), and *Lagerstroemia speciosa* (LsFME) through acetic acid-induced writhing test.

Group	Dose (mg/kg body weight)	Number of writhing	% of inhibition
DrFME	200	29.40 ± 5.20	31.20
	400	19.80 ± 3.27	51.20
CfFME	200	21.40 ± 3.78	46.20
	400	14.40 ± 2.60	64.34
LsFME	200	33.20 ± 3.30	17.44
	400	27.40 ± 5.17	32.63
**Positive control (Aspirin)**	200	11.80 ± 3.11	74.72
**Negative control (Distilled water)**	10 ml/kg body weight	42.00 ± 8.15	0

### 3.9 In vivo antidiarrheal study (castor oil-induced diarrheal test)

In the *in vivo* antidiarrheal study, castor oil was used to induce diarrhea in mice, and then extracts were administered to see the inhibition of diarrhea. The results are displayed in Table 5. From the result, all the extracts showed good antidiarrheal activity, while DrFME served as the best among them. The doses of DrFME (200 and 400 mg/kg body weight) dose-dependently reduced the total number of diarrheal feces. The percentage inhibition of diarrhea was 70.27% and 46.15% respectively. Among the other treatments with LsFME (400 mg/kg body weight) and CfFME (400 mg/kg body weight) showed inhibition (%) which are 61.27% and 57.67% respectively. Loperamide hydrochloride was administered as a positive control which achieved 85.39% inhibition of diarrhea.

**Table 5:** *In vivo* antidiarrheal activity of methanolic extracts of flowers from *Delonix regia* (DrFME), *Cassia fistula* (CfFME), and *Lagerstroemia speciosa* (LsFME) through castor oil-induced diarrheal test.

Group	Dose (mg/kg body weight)	Number of diarrheal feces	% of inhibition of diarrhea
**DrFME**	200	6.80 ± 1.30	37.21
	400	4.60 ± 1.40	57.67
**CfFME**	200	6.40 ± 1.34	40.15
	400	4.20 ± 1.30	61.27
**LsFME**	200	5.80 ± 0.84	46.15
	400	3.20 ± 0.84	70.27
**Positive control (Loperamide hydrochloride)**	3.0	1.60 ± 0.55	85.39
**Negative control (Distilled water)**	10 ml/kg body weight	10.80 ± 0.84	0

### 3.10 In vivo antihyperglycemic study (oral glucose tolerance test)

Administration of the extracts to the glucose-loaded mice resulted in a dose-dependent reduction in the levels of blood glucose. The results are shown in Table 6. The highest activity was shown by LsFME, where at doses of 200 and 400 mg/kg body weight, lowering of blood glucose levels in experimental mice were 26.8% and 46.94% respectively, compared to the lowering (53.95%) in mice by the positive control Glibenclamide. Among the other treatments with DrFME (400 mg/kg body weight) and CfFME (400 mg/kg body weight) showed inhibition (%) which are 16.60% and 12.50% respectively.

**Table 6:** *In vivo* antihyperglycemic activity of methanolic extract of flowers from *Delonix regia* (DrFME), *Cassia fistula* (CfFME), and *Lagerstroemia speciosa* (LsFME) through oral glucose tolerance test.

Group	Dose (mg/kg body weight)	Blood glucose level (mmol/L)	% lowering of blood glucose level
**DrFME**	200	6.38 ± 0.15	6.67
	400	5.72 ± 0.13	16.60
**CfFME**	200	6.46 ± 0.25	5.83
	400	6.00 ± 0.28	12.50
**LsFME**	200	5.02 ± 0.13	26.80
	400	3.64 ± 0.29	46.94
**Positive control (Glibenclamide)**	10	3.16 ± 0.20	53.95
**Negative control (Distilled water)**	10 ml/kg body weight	6.86 ± 0.12	–

## 4 Discussion

The phytochemicals or bioactive compounds are responsible for numerous health-promoting activities. Advances in medicinal plant research have opened new frontiers in the search for effective medicines from natural sources. That's why plants are continuously being explored for their chemical diversity. In our study, the presence of a wide range of phytochemicals supported the medicinal effects they have provided.

Quantifying the phytochemicals such as total phenolic, flavonoid and tannin contents serve as the background information for some of the bioactivities. Previously many studies claimed that phenolic compounds are one of the responsive factors for antioxidant activity of medicinal plants [40]. Flavonoids are also physiologically active constituents that have been traditionally utilized to treat different human diseases especially cardiovascular disease [41]. Tannins are and attributed to antioxidant, antimicrobial, anti-mutagenic, and other health-promoting activities [42]. Therefore, as evident from our study, the presence of a high quantity of phenol as well as flavonoid and tannin can be credited as contributing factors to the medicinal values of these flowers. Shabir et al. reported the total phenolic and total flavonoid content in 80% methanolic extract of *Delonix regia* flower as 2.24 g/100 g dry extract and 0.81 g/100 g dry extract respectively [5]. P. Siddhuraju et al. reported the total phenolic content of 90% methanolic extract of *Cassia fistula* flower as 6.52 g/100 g extract [43]. Irshad et al. reported total phenolic and flavonoid content of *Cassia fistula* fruit pulp extract as 114.23 mg/g and 74.65 mg/g respectively. They also quantified the total phenolic and flavonoid content in seed extracts as 109 mg/g and 67.78 mg/g respectively [18]. A study conducted by Kesavanarayanan et al. reported the total phenolic, flavonoid, and tannin content in methanolic extract of leaves of *Lagerstroemia speciosa* as 300.11 μg/mg, 53.12 μg/mg and 118.90 μg/mg respectively [44].

Many diseases are associated with oxidative stress and antioxidants are responsible for inhibiting the harmful effects of oxidative stress [45]. Natural antioxidants give protection to the human body from free radicals and thereby help to protect from the progression of many diseases [46]. Shabir et al. found the IC50 value of 80% methanolic extract of *Delonix regia* flower as 14.80 μg/mL in the DPPH radical scavenging assay [5]. Irshad et al. reported the EC50 value of methanolic extract of *Cassia fistula* fruit pulp and seed extract as 0.915 and 1.088 mg/mL in the DPPH radical scavenging assay. Tiwari et al. found the IC50 value of 80% methanolic extract of *Lagerstroemia speciosa* flower as 3.23 μg/mL in the DPPH radical scavenging assay [47]. While some of the previous studies reported the antioxidants activity of different plant parts of these plants, the present study also validates the utility of flowers as a source of antioxidants.

Parasitic worms are responsible for some of the most widespread chronic human infections. These infestations are more prevalent in underdeveloped areas with poor people where children are more susceptible than adults [48]. All the extracts performed very well in terms of anthelmintic activity which suggests their further broad-spectrum analysis with multiple targets. Our current study indicates that LsFME has been found to possess anthelmintic potential in a dose-dependent manner. This activity may be attributed to the presence of a high amount of phytochemicals it possesses. Phytoconstituents such as alkaloids, tannins, phenols, etc. May be responsible for the activity. Alkaloids cause paralysis of worms by acting on the central nervous system (CNS) whereas tannins and polyphenols selectively bind to free proteins present in the gastrointestinal (GI) tract and eventually cause mortality. On the other hand, the anthelmintic efficacy of saponins is due to its membrane permeabilizing property [49]. Previously, Ahirrao et al. reported that aqueous and methanolic extracts of the *Delonix regia* flower had good anthelmintic activity against earthworm *Pheritima posthuma* [50]*.* Irshad et al. reported the anthelmintic property of the methanolic seed and pulp extracts of *Cassia fistula* [51]*.* The present study showed consistency with previous findings and provided novel reporting of activity against aquarium worm *Tubifex tubifex*.

Formation of unusual blood clots in the arteries and veins is called thrombosis which is a contributor to cardiovascular complications. There are several drugs available in the market to dissolve the blood clots [52]. In the present study, DrFME showed remarkable thrombolytic activities. The other two extracts also showed good results. Previous studies reported the cardioprotective effect of the *Delonix regia* leaf extract where it exhibited a vasodilation effect on heart vessels [6]. While thrombolytic agents are used to dissolving blood clots, arterial and venous thrombotic disorders are treated by the anticoagulants. It is worth mentioning that, drug therapy for the dissolution of blood clots is the first-line approach while inhibition of the intrinsic and extrinsic pathways in the blood clotting cascade is used in acute and intensive care approaches. These two therapeutic approaches are not identical [53]. The PT and aPTT tests are popular tests for monitoring coagulation and anticoagulant therapy where PT assay monitors the extrinsic coagulation pathway and the aPTT test is concerned with the intrinsic coagulation [54]. In addition to the thrombolytic activity, DrFME also showed remarkable anticoagulant activities. The thrombolytic and anticoagulant potential of *Delonix regia* flower extracts further validate its ethnomedicinal importance. Previously, the thrombolytic potential of some medicinal plants such as *Averrhoa bilimbi, Clerodendrum viscosum, Drynaria quercifolia, Trema orientalis, Bacopa monnieri, Capsicum frutescens, Brassica oleracea, Urena sinuata, Geodorum densiflorum, Pistia stratiotes, Smilax zeylanica, Pandanus foetidus, Tabernaemontana coronaria* etc. has been explored and reported in Bangladesh [[55], [56], [57]]. The present study also demonstrated that the flower extracts had potent clot lysis activity very similar to the results previously observed.

Compounds with potent biological activity may not be utilized in pharmacological preparations if they showed hemolytic effects. Hemolytic assays, therefore, symbolizes a useful starting point by providing the baseline information on the interaction between bioactive compounds and biological entities at the cellular level. Hemolytic activity displayed by the compounds suggests general cytotoxicity towards normal healthy cells. Also, they indicate potential cytotoxicity [58]. The hemolytic activity of the flower extracts utilized in the present study was negligible as they showed an IC50 value greater than 200 μg/mL [59], which indicated the less detrimental effect of their pharmacological applications.

Pain is a component of the immune system, serves as a protection to damage caused by certain stimuli, and also provides a symptom of many diseases. It often causes discomfort and requires treatment with analgesics [60]. Acetic acid-induced writhing tests in mice is a model of visceral pain that is highly sensitive and useful for screening analgesic drugs. However, it only represents pain sensation by triggering a localized inflammatory response [61]. Ezeja et al. achieved 63% inhibition at 400 mg/kg body weight dose in the acetic acid-induced writhing test by methanolic extract of *Delonix regia* leaves [62]. Patwardhan et al. studied the analgesic activity of the ethanolic extract of *Cassia fistula* leaves and barks where they achieved 21.73% and 29.34% inhibition respectively at 400 mg/kg body weight dose [63]. Aqueous ethanolic extract of *Lagerstroemia speciosa* leaves provided 62.78% pain inhibition at 400 mg/kg body weight dose in a study conducted by Gupta et al. [64].

Diarrhea is one of the most common infectious diseases prevalent mainly among poor communities [38]. Castor oil-induced diarrheal test is a widely employed experiment where castor oil works by changing the permeability of the intestinal mucosal membrane to electrolytes and water and thus produces diarrhea [65]. The antidiarrheal activity of plant extract is often attributed to the presence of tannins that form protein tannate to denature proteins. It creates resistance in the intestinal mucosa which reduces secretion [66]. The presence of high content of tannins in our study thus may support the excellent antidiarrheal activity by the flower extracts. Rajabhau et al. found 29.76% and 70.23% protection against diarrhea at the 250 and 500 mg/kg body weight dose respectively by ethanolic extract of *Delonix regia* flowers [67]. According to the study of Rahman et al., dried fruits of *Lagerstroemia speciosa* showed significant antidiarrheal activity [68]. Another study conducted by Hussain et al. reported that methanolic extract of the root of *Lagerstroemia speciosa* at the dose of 200 and 400 mg/kg body weight exhibited a reduction of diarrheal feces by 32.75% and 51.72% [26].

Diabetes is a chronic metabolic disease. In the case of type 1 diabetes, the body is unable or inadequately produce insulin while in the case of type 2 diabetes, insulin is less prevalent in the bloodstream or less properly utilized. High blood sugar levels lead to a condition called hyperglycemia when insulin is not provided to utilize glucose diabetes [69]. The LsFME resulted in a decrease in blood glucose levels more readily than others. This may be because of the amount of flavonoids and phenols it posses. Flavonoids have been studied concerning diabetes mellitus, either through the inhibition of intestinal α-glucosidase enzyme or through their capacity to avoid glucose absorption and/or to improve glucose tolerance [70]. In addition to that, previous studies reported the efficacy of phenols as efficient antihyperglycemic agents [71]. Previously, Rahman et al. reported significant glucose-lowering capacity exhibited by methanolic extract of *Delonix regia* leaves [7]. Antihyperglycemic activity in glucose-overloaded hyperglycemic rats by petroleum ether (60–80°), chloroform, acetone, ethanol, aqueous, and crude aqueous extracts and two fractions of ethanol extract obtained from the flowers of *Cassia fistula* has been reported by Jarald et al. [72]. Saha et al. found a significant blood sugar level lowering effect by the hot water extract of *Lagerstroemia speciosa* leaves [73].

## 5 Conclusions

Our present study revealed that the flowers from all of the three selected plants are rich in phytochemicals. The study also revealed the potential bioactivities of the methanolic extracts of three flowers. DrFME showed good thrombolytic and anticoagulant activity, CfFME showed good analgesic activity and LsFME showed good antioxidant, antihelminthic, antidiarrheal, and antihyperglycemic activity. There are previous reports of utilizing different plant parts of these plants while the use of flowers were less focused. Our study not only explored the potential medicinal properties of these flowers but also indicates that flowers can also be a source for compounds with potential pharmacological properties. It can be said that these flowers hold promises for their future utilization in a more elaborated and targeted way. The present study is a baseline foundation for the exploration of eficacy of the flower extracts. The toxicity and safety of the extracts should be evaluated in future endevours. Further investigations should be directed at the isolation and characterization of specific compounds responsible for specific bioactivity in the background of the elucidation of their mechanism of action. Moreover, the compounds can be studied elaborately to assess the possibility of developing novel pharmaceutical agents of plant origin to fight against diseases and disorders.

## Funding

This research was partially supported by the research grant provided by GARE (Grant for Advanced Research in Education Grant ID-LS2016165 and Memo No. 37.20.0000.004.033.020.2016.7725) funded by the 10.13039/501100004567Ministry of Education, Bangladesh and 10.13039/501100008804Special Allocation in Science and Technology of Ministry of Science and Technology (Memo No. 39.00.0000.09.06.79.2017/ES-99), Bangladesh.

## Credit author contribution statement

**Faisal Bin Rahman:** Conceptualization, Methodology, Software, Validation, Formal analysis, Investigation, Data curation, Writing - original draft, Preparation, Writing - review & editing, Visualization. **Sium Ahmed:** Conceptualization, Methodology, Software, Validation, Data curation, Writing - original draft, Preparation, Writing - review & editing, Visualization. **Priya Noor:** Validation, Formal analysis, Investigation, Investigation. **Mir Md Mahbubur Rahman:** Formal analysis, Investigation. **S.M. Azimul Huq:** Formal analysis, Investigation. **Md Taharat Elahi Akib:** Formal analysis, Investigation. **Abdullah Mohammad Shohael:** Conceptualization, Resources, Writing - review & editing, Supervision, Project administration, Funding acquisition.

## Declaration of competing interest

The authors declare no conflict of interest.
